# A conceptual framework for capacity strengthening of health research in conflict: the case of the Middle East and North Africa region

**DOI:** 10.1186/s12992-019-0525-3

**Published:** 2019-11-28

**Authors:** Nassim El Achi, Andreas Papamichail, Anthony Rizk, Helen Lindsay, Marilyne Menassa, Rima A. Abdul-Khalek, Abdulkarim Ekzayez, Omar Dewachi, Preeti Patel

**Affiliations:** 1 Conflict Medicine Program, Global Health Institute, American University of Beirut, Beirut, 1107 2020 Lebanon; 2 School of Politics & International Relations, Queen Mary University of London, London, E1 4NS UK; 3 Department of War Studies, King’s College London, London, WC2R 2LS UK

**Keywords:** Capacity strengthening, Conceptual framework, Research, Health, MENA, Conflict

## Abstract

**Background:**

In conflict settings, research capacities have often been de-prioritized as resources are diverted to emergency needs, such as addressing elevated morbidity, mortality and health system challenges directly and/or indirectly associated to war. This has had an adverse long-term impact in such protracted conflicts such as those found in the Middle East and North Africa region (MENA), where research knowledge and skills have often been compromised. In this paper, we propose a conceptual framework for health research capacity strengthening that adapts existing models and frameworks in low- and middle-income countries and uses our knowledge of the MENA context to contextualise them for conflict settings.

**Methods:**

The framework was synthesized using “best fit” framework synthesis methodology. Relevant literature, available in English and Arabic, was collected through PubMed, Google Scholar and Google using the keywords: capacity building; capacity strengthening; health research; framework and conflict. Grey literature was also assessed.

**Results:**

The framework is composed of eight principal themes: “structural levels”, “the influence of the external environment”, “funding, community needs and policy environment”, “assessing existing capacity and needs”, “infrastructure and communication”, “training, leadership and partnership”, “adaptability and sustainability”, and “monitoring and evaluation”; with each theme being supported by examples from the MENA region. Our proposed framework takes into consideration safety, infrastructure, communication and adaptability as key factors that affect research capacity strengthening in conflict. As it is the case more generally, funding, permissible political environments and sustainability are major determinants of success for capacity strengthening for health research programmes, though these are significantly more challenging in conflict settings. Nonetheless, health research capacity strengthening should remain a priority.

**Conclusion:**

The model presented is the first framework that focuses on strengthening health research capacity in conflict with a focus on the MENA region. It should be viewed as a non-prescriptive reference tool for health researchers and practitioners, from various disciplines, involved in research capacity strengthening to evaluate, use, adapt and improve. It can be further extended to include representative indicators and can be later evaluated by assessing its efficacy for interventions in conflict settings.

## Key messages

- Health research capacity strengthening is vital, especially in conflict areas.
- Frameworks for health research capacity strengthening in conflict settings are almost non-existent.
- The MENA region is in urgent need of capacity strengthening of health research given that it only contributes to 1.5% of annual global publications.
- The model developed here will be useful for interdisciplinary health and development researchers and practitioners in academia and humanitarian and development NGOs.

## Background

Recent reports have estimated that almost two billion people currently live in areas of conflict and fragility and the world is experiencing the largest refugee crisis since the Second World War, with a new record high of 70.8 million forcibly displaced people worldwide in 2019 [1].

The Middle East and North Africa (MENA) region, which is the focus of this paper, is witnessing an unprecedented scale of multiple and complex emergency situations with protracted conflict being a pervasive reality in the region (see Table 1 for definition). For example, the Syrian, Yemeni and Libyan crises that started in 2011, the earlier Iraqi (since 2003) and Palestinian (since 1948) conflicts along with other emerging conflicts such as in Sudan (in 2019) continue to affect the whole region [6–9]. Moreover, following the Syrian crisis, some 6.6 million Syrians have been internally displaced and another 5.6 million are registered as refugees in the surrounding countries [10]. As for Yemen, more than 2 million people have been displaced and 14 million are currently facing pre-famine conditions [11]. The situation in both Iraq and Libya is also still unstable with continued insecurity [12]. The region is thus facing various health problems, such as the re-emergence of communicable diseases like polio and malaria, a rise in malnutrition, post-traumatic stress disorder (PTSD), the changing patterns of multidrug resistant pathogens, and the burden of various already existing communicable and non-communicable diseases; all of which need to be urgently addressed [13].

**Table 1:** Key definitions

***Protracted Conflict:*** Hostile interactions which extend over long periods of time with sporadic outbreaks of open warfare fluctuating in frequency and intensity [2].	
***MENA (Middle East and North Africa) Region:*** Covers 24 countries, namely the 21 members of the Arab League (Algeria, Bahrain, Djibouti, Egypt, Iraq, Jordan, Kuwait, Lebanon, Libya, Mauritania, Morocco, Oman, Palestine, Qatar, Saudi Arabia, Somalia, Sudan, Syria, Tunisia, the United Arab Emirates and Yemen), as well as Iran, Israel and Turkey [3, 4].	
***Capacity strengthening:*** As a working definition, capacity strengthening can be understood as a process of developing, upgrading and/or expanding pre-existing capabilities at the individual, organisational, and institutional levels to plan, conduct, and disseminate evidence-based knowledge [5].	

While research capacity strengthening might seem counterintuitive in these conflict settings, where the immediate pressures of survival and relief from conflict become the dominant paradigm, we argue that as crises are increasingly protracted, a different response paradigm is needed, that includes research capacity strengthening. Doing so could aid in collecting evidence of the highest standard, assessing actual health needs of affected populations, bridging the gap between research and practice and eventually informing advocacy and policy change [14]. Additionally, strengthening research capacity could help address major endemic diseases in their epidemiological context, evaluate and improve relief work, and support social changes to improve the accountability and quality of assistance provided in these settings and thus enhance the wellbeing of affected communities. This would also help influence post-conflict policies and perhaps even promote efforts for peace [15]. It is with this understanding that this paper seeks to develop a framework for capacity strengthening for health research in conflict, with a specific focus on the MENA region.

### The challenges of research capacity strengthening in MENA and beyond

Research capacity strengthening has become a growing priority for both local and regional institutions in low- and middle-income countries (LMICs), as well as for international organisations and research funding agencies [16, 17]. This has been due, in part, to the need to implement evidence-based interventions in LMICs, where health research is limited [18, 19], as evidence-based research is vital for improving health outcomes, systems, and policies [20]. While there is widespread acknowledgment of the need to improve research outputs from LMICs, defining what capacity strengthening for health research entails is both conceptually complex and context-sensitive [21–24] (see Table 1 for our definition).

One of the major challenges of research capacity strengthening interventions in LMICs and MENA is funding and investment [18]. Despite becoming subject to new health, social care and development challenges, the MENA region’s health research is fragmented and inadequate due to the lack of national policies or strategic plans that favour the investment in such projects [25]. Though the region is rich in oil and includes high-income economies, investment in research in the MENA region is lagging behind at the global scale. For example, despite having 5% of the world’s population, the region contributes only 1.5% of the scientific papers published globally every year [26, 27]. This deficit is further evidenced when considering the expenditure on research and development, as percentage of Gross Domestic Product (GDP), where the whole MENA region allocates an average of 0.93% of its GDP compared to the global average of 2.23% GDP [28]. Meanwhile, a recent narrative review found that among reported health research capacity strengthening interventions in LMICs between 1992 and 2017, only four studies (9% of interventions) were in the MENA region, and focusing only on the United Arab Emirates, Qatar, Iran and Turkey [24].

When it comes to conflict-specific areas, however, conducting research and capacity strengthening faces serious challenges and constraints. The priorities of international, national and local interventions shift between security, conflict resolution, acute humanitarian response and refugee and migration management. In the context of prolonged conflict, such as that experienced by some MENA countries, institutions partially resume their pre-conflict activities in the context of continuing and emerging challenges, which include lack of security, collapse of infrastructure, and the need to cultivate trust among stakeholders involved in research. These challenges are usually prioritised over research investments, funding and related activities [29–31].

Despite the constraints, there is a growing interest in research capacity strengthening interventions in the region [7]. Health researchers from the MENA have begun to explore solutions for research capacity deficits in the context of crisis in collaboration with international partners. There are some leading examples of such interventions: such as the European Union funded Research Capacity for Public Health in the Mediterranean (RESCAP-MED) collaboration which focused on capacity building for non-communicable disease research in the MENA region since the Arab revolutions unfolded in 2010 [32, 33]. A similar partnership is the RECAP project that brings together the London School of Tropical Medicine (LSHTM), the American University of Beirut, the University of Sierra Leone, as well as non-governmental organizations (NGOs), with the aim of strengthening research capacity and the preparedness for humanitarian crises and epidemics [34]. Research for Health in Conflict MENA (R4HC-MENA) – the project that this research forms a part of – is another partnership between UK-based and MENA academic institutions, that aims to develop sustainable research capacity in the region to address major health challenges arising from conflict, with a focus on Jordan, the Occupied Palestinian Territories, Lebanon and Turkey, as well as conflict-affected populations in Syria, Iraq, Libya and Yemen [35].

Much of the literature on research capacity strengthening explores practical ways of strengthening the translation of research into policy and practice [36–39]. While this is fundamental for research capacity strengthening, here we are interested in exploring conceptual frameworks that focus on strengthening research capacity itself as well as implications for evidence-based policy and practice [5, 40–45]. Most conceptual frameworks thus far have been developed by international and academic institutions to define and assess capacity strengthening for health research in general settings. They often provide guidance and structure on evaluation, sustainability, administration, and data sharing and communication [46–48] but never target the specificities of conflict settings.

Thus, building on the work of Marjanovic et al. which highlighted the importance of research capacity building in protracted conflicts [49], the aim of this paper is to synthesise a framework for health research capacity strengthening in conflict in the MENA region that provides a better understanding of contextualized capacity strengthening interventions. The framework builds on the analysis of existing models and frameworks for health research capacity strengthening in LMICs and contextualises them into an operational framework for capacity strengthening for the region and beyond. More specifically, the paper seeks to reformulate the existing frameworks where security and functional policy environments have been taken for granted. The framework is intended to be a stimulus (and a working tool) for further strengthening of infrastructures for empirical research in conflict, where evidence-based service provision and quality of care research are often lacking.

## Results

Our search found very limited English-language literature on capacity strengthening of health research in conflict settings, despite its importance in bringing innovative health research findings to humanitarian and frontline practitioners [14]. DeJong and colleagues [50] have highlighted the capacity strengthening implications of building research networks that elevate the voice of internationally underrepresented regions from conflict settings and their contributions to setting research priorities. Meanwhile, the context-specific requirement for capacity strengthening priorities in emergency settings has been addressed in reviews on capacity strengthening by Eade [51] and by Woodward et al. [30]. For the most part, operational research has mainly been conducted by non-governmental organizations [24, 52, 53]. Moreover, related literature in Arabic was almost non-existent, with most of what was found focusing on the lack of research culture in the region despite its substantive financial and human resources [54–56].

In terms of frameworks, we found 17 research capacity building/strengthening frameworks. The frameworks are diverse in terms of both region/country of intervention and of the themes targeted. A summary of the major, but not all, themes targeted in these frameworks is represented in Table 2.

**Table 2:** Summary of selected capacity strengthening frameworks

Reference	Themes	Location
[57–60]	Partnership, training, leadership, mentorship	USA [57], Canada [58], African continent [60]
[61] [62] [39]	Policy	LMICs [39], Bangladesh, Fiji, India, Lebanon, Moldova, Pakistan, South Africa, Zambia [61], Bangladesh, Gambia, India and Nigeria [62]
[63]	Funding	Bangladesh
[64, 65]	Bridging with practice	Australia
[20, 40, 41, 44, 66–70]	Multiple themes: financing, sustainability, political environment, mentorship, training …	Multiple

However, all of these frameworks were applied for interventions taking place in various places like the UK, Europe, Bangladesh, Australia, and Canada but did not target the MENA region or any conflict-affected setting specifically. Websites of organisations working in conflict-affected areas in the MENA region, such as Oxfam, Médecins Sans Frontières (MSF), International Development Research Centre (IDRC), Norwegian Refugee Council (NRC) and Danish Refugee Council (DRC) were also assessed. Although they provide operational research, training and capacity strengthening initiatives, we could not find comprehensive research strengthening frameworks which they adopt or use on their websites.

Based on the aims of our work, we found two frameworks to be particularly relevant, especially as they provide indicators for the evaluation of each theme within their frameworks which can be re-evaluated during the lifetime of the R4HC-MENA project. For instance, Cooke’s [40] framework was used for interventions in complex settings by Marjanovic et al. [49]. Meanwhile, DFID’s [41] model provides a detailed “how to note” that describes the major steps needed to be taken to build research capacity at the individual, institutional and organisational levels while including the main criteria that should be taken into account for the evaluation of the progress at each of these levels. The model does not address the specificities of conflict nor that of the MENA region but is rather more generic. A summary of both frameworks is presented below. However, this is not to discount the importance of the other selected frameworks (cited above) which were also taken into consideration in guiding us in the synthesis of the proposed framework.

**Fig. 1:**
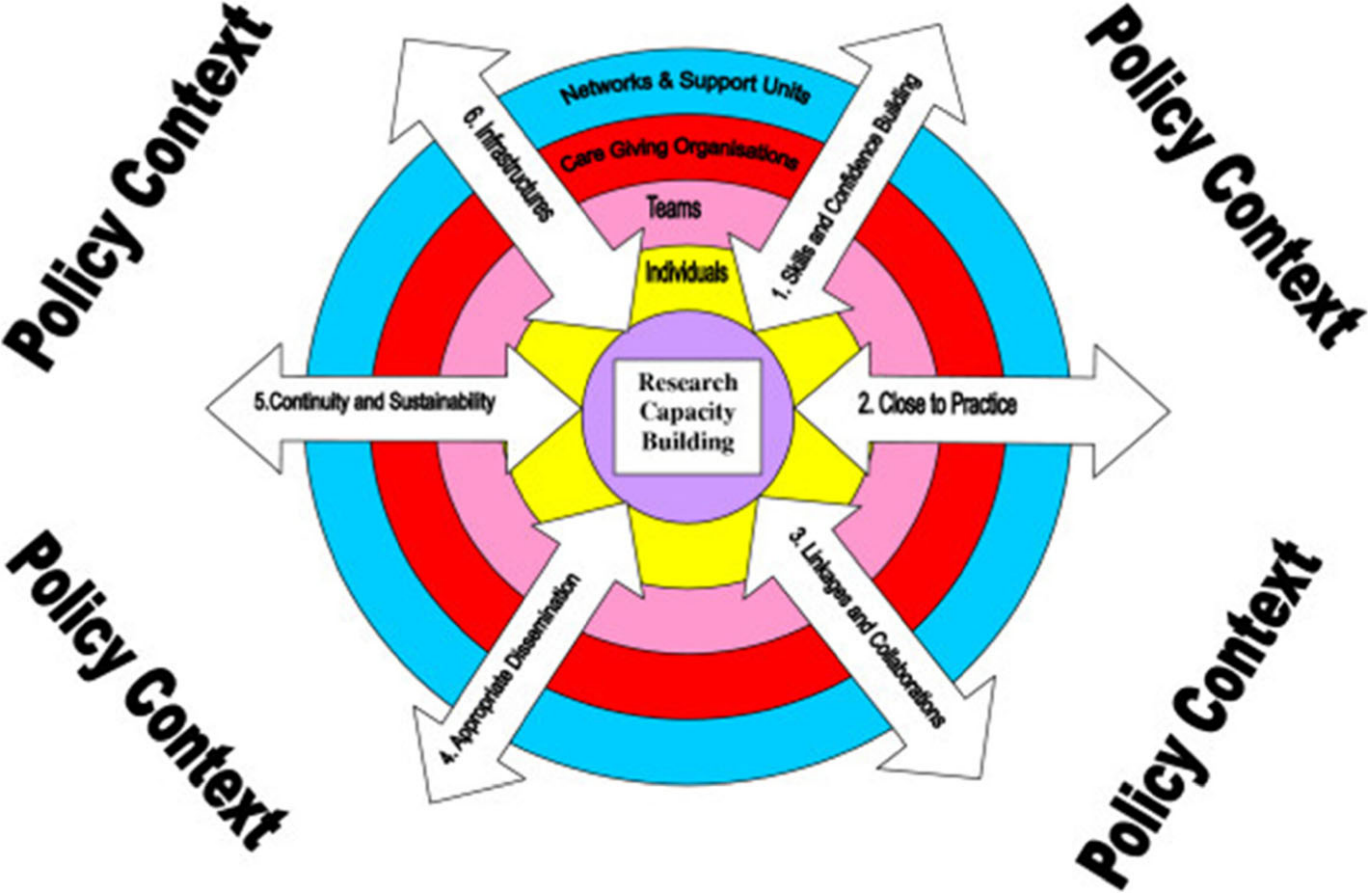
Cooke's Capacity building of health research framework [40]

Cooke’s framework focuses on the implementation and evaluation of capacity strengthening of health research (Fig. 1). One of the important factors it addresses is that capacity strengthening can be considered as both “an end” and as a “process to an end.” The framework has two dimensions: four structural levels of development and six principles of capacity strengthening that cut across the four structural levels. The structural levels include individuals, teams, institutions and networks. The six principles are: building skills and confidence, developing linkages and partnerships, ensuring the research is ‘close to practice’, developing appropriate dissemination, investments in infrastructure and building elements of sustainability and continuity [40]. Cooke’s framework aims to bridge the gap between research and practice by involving practitioners in primary research. Moreover, Cooke highlights the impact of policy on research capacity strengthening and how it can strongly influence opportunities to develop, support and sponsor research and researchers. Nonetheless, when exploring published journal articles that focus on health research capacity strengthening, Cooke’s framework was highly cited between 2005 and 2019 (254 times), in a field which is not widely studied.

**Fig. 2:**
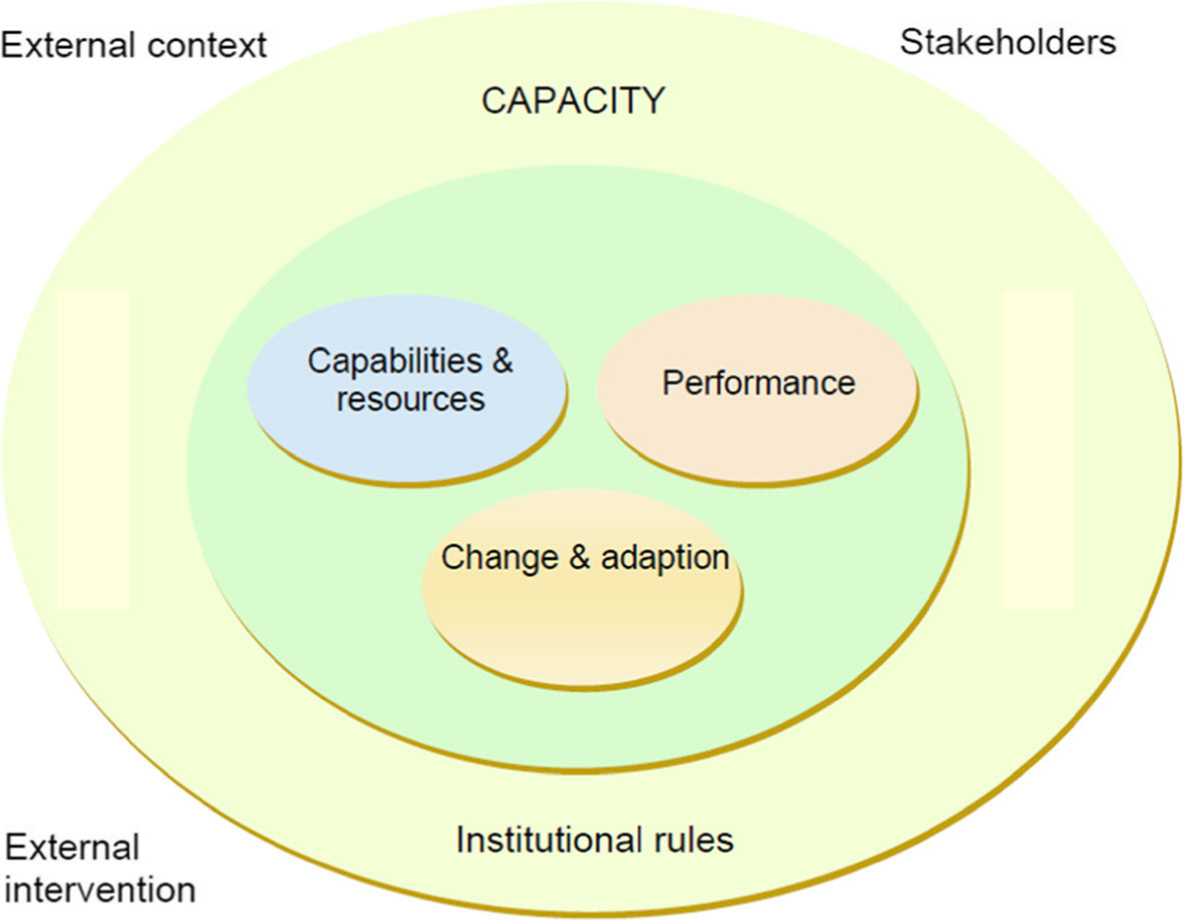
DFID’s key elements of capacity building [41]

DFID’s framework sets out a step-by-step approach to how capacity strengthening can be addressed, implemented and evaluated (Fig. 2). It provides definitions of concepts and includes good practice examples of capacity strengthening, such as the partnership between DFID and the Southern African Federation of the Disabled (SAFOD) in implementing the SAFOD Research Programme (SRP) that aims to strengthen the capacity of researchers with disabilities by training them to be fully involved in research [71]. The major aspects included in this framework are performance, change and adaptation, and capabilities and resources. DFID also adopts the European Centre for Development Policy Management’s core capabilities: empowerment, management of relationships, implementation of goals, resource mobilisation, managing change, encouraging motivation and managing complexity. Based on these capabilities, capacity strengths and weaknesses can be diagnosed in order to be improved over time.

However, the available frameworks have been the subject of critique from many researchers in this field. Bates et al. [18], Cole et al. [72] and Marjanovic et al. [49] have analysed the different aspects of the available conceptual frameworks for capacity strengthening and concluded that these frameworks and their corresponding indicators do not focus on interlinking the different activities with resulting outputs and outcomes at the different structural levels, and thus lack cohesion and consistency [73]. Cooke’s framework – like many others – is not able to fully cover the challenges of research capacity strengthening in conflict-affected settings. Moreover, and as indicated by Cooke herself, the framework’s indicators are not exhaustive as most of them focus on capacity strengthening as an end; other indicators should be included that take into account that the desired outcomes of research capacity strengthening should be directly related to social impact.

Despite its detailed description and being the most comprehensible among the non-academic frameworks, DFID’s framework also needs to be tailored to conflict settings, along with a stronger outcome-oriented approach. Indeed, Marjanovic et al. [49] highlight the limited literature on using frameworks for capacity strengthening in settings of prolonged conflict. All told, there is a clear need to develop new frameworks for capacity strengthening of health research.

### A conceptual model for capacity strengthening of health research in conflict

**Fig. 3:**
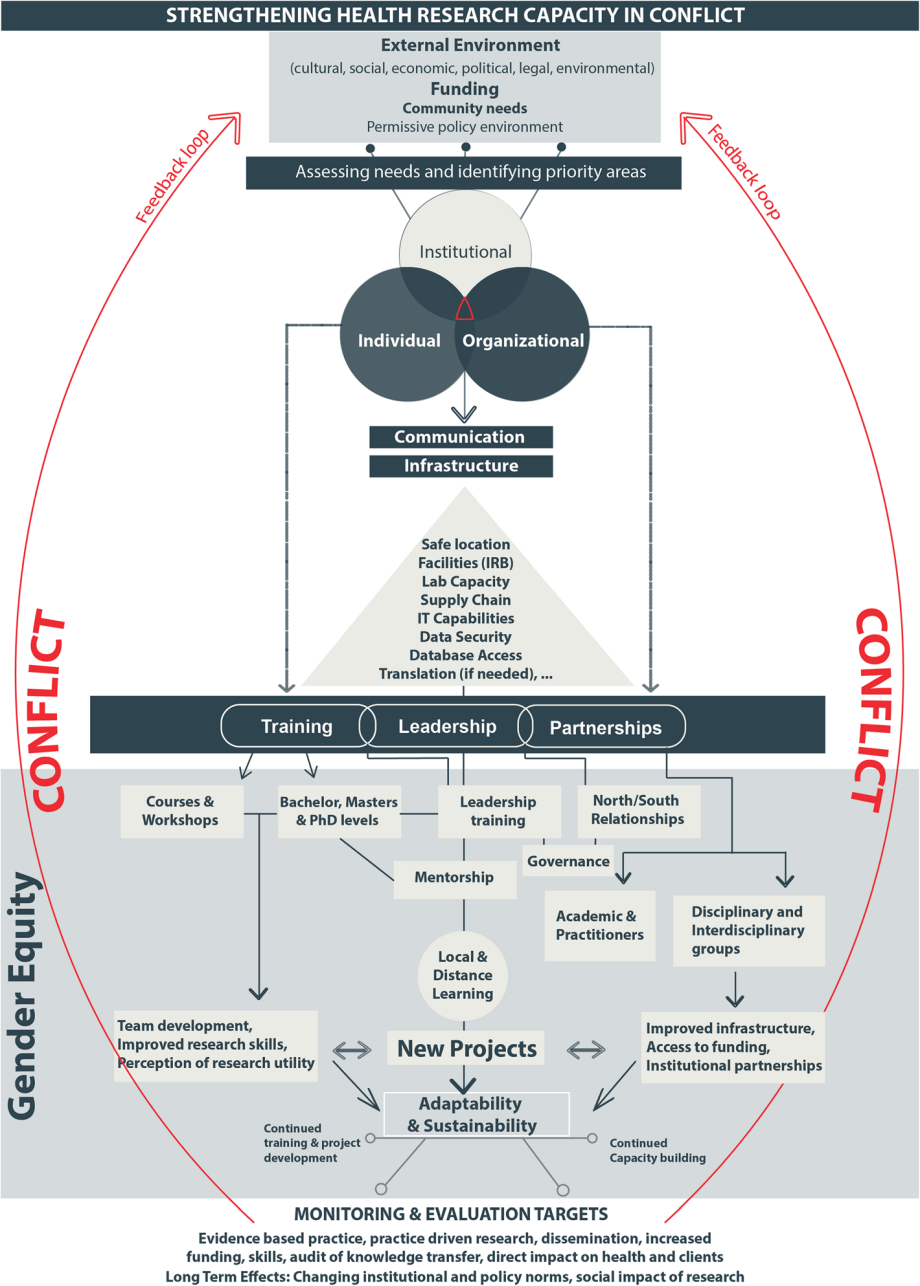
Proposed conceptual framework for health research capacity strengthening in conflict

Our analysis of the available research capacity strengthening frameworks has highlighted a significant gap in targeting conflict-affected areas, including the MENA region. Thus, as a start, the proposed framework seeks to address this gap, and tries to account for the factors and levels we believe are elemental to research capacity strengthening in the MENA region more broadly, and in conflict-affected areas specifically. As such, the proposed framework recognises major factors that contribute to enhancing research capacity strengthening in conflict settings, at the institutional, organisational and individual levels with a view to producing research that has social impact. Moreover, highlighting factors like communication, infrastructure and adaptability, which are problematic in conflict, contextualises the framework to conflict-affected areas. The proposed conceptual model for health research capacity strengthening in conflict-affected areas in the MENA region is presented in Fig. 3 below. This is followed by a more detailed explanation, with reference to the literature and the specificities of conflict-affected areas in the region, of each of the eight major elements within this framework which are: structural levels, external environment, funding, community needs and policy environment, assessing existing capacity and needs, infrastructure and communication, partnership, training and leadership including gender equity, adaptability and sustainability, and monitoring and evaluation.

## Discussion

### Structural levels

One of the main areas of disagreement in the capacity strengthening literature is the classification of the structural levels involved in research capacity strengthening. How these levels are structured and the interconnections between them determine how strategies for capacity strengthening are designed [74, 75]. The framework of Levels and Dimensions that emerged in the mid-1990s indicated that any capacity strengthening initiative should be examined from all of the proposed structural levels [76]. This framework is extensively applied; however, the specific structural levels used vary widely based on the organisation. For instance, the most commonly used structural levels of capacity strengthening, adopted by the United Nations Development Program (UNDP) and DFID, are the individual, institutional and organisational levels [41, 74]. The individual level includes researchers and research teams; the organisational level is composed of university research departments, think tanks and similar organisations; and the institutional level encompasses the regulatory environment and includes governmental bodies and policy makers [74]. Minor modifications to the classification of the structural levels listed above also exist in the literature. For example, LaFond and Brown [43] call these levels personnel, organisation and system, and in Cooke’s [40] framework, the individual level is divided into individuals and teams, and the institutional level is synonymous with the term networks [77, 78]. Meanwhile, Lansang and Dennis [69] add two additional levels: national and supranational, to address investments in capacity strengthening at the national and international levels. Bates, Boyd [18] combine the organisational and institutional levels together under the term “institutional” and add a societal level – similar to the “national” level of Lansang and Dennis’ work – while keeping the individual level that included researchers and research groups. The WHO, however, adopts a framework that has five structural levels that take into account the complexity of the public sector: individual, organisational, networks, institutional context of the public sector and action environment are differentiated from each other [76, 79].

Higher levels of categorisation can lead to unnecessary complexity in conflict settings. In our framework, we decided to adopt the three major levels used by DFID and UNDP: individual, organisational and institutional. However, we mainly focused on exploring the major themes and sub-themes that are related to research capacity strengthening at the individual and organisational levels since, as mentioned earlier, in times of conflict it is more than challenging to have interventions at the institutional level (off-white in Fig. 3) as defence, security and immediate humanitarian response are prioritised over research [30, 80].

### External environment

In contexts of prolonged conflict, the external environment is an overarching factor in successful health research capacity strengthening, and should be the point of departure in thinking about the health research capacity strengthening process. Drawing on LaFond and Brown [43], we have used the following six factors: cultural, social, economic, political, legal, and environmental as issues to consider in the overall environment. This conceptualisation of the external environment is also seen in the DFID framework, which places external context and external intervention in the outermost sphere of influence [41]. Similarly, White’s framework also considers the social and cultural environment as the major determinants of research on health [44]. While the external environment certainly plays a role in any capacity strengthening project, it is particularly important in conflict settings, as each of the aspects of the external environment is affected by conflict.

Furthermore, the political and legal situation may be in a state of flux due to conflict that also makes the cultural and social aspects hard to identify due to the complexity and the variability in these determinants among affected populations. Therefore, the environmental situation with regards to the six factors above can greatly advance or delay health research capacity strengthening, *especially* in conflict [14, 80]. For instance, a recent study conducted by Al-Hamadani et al. [81] stated that the historical, ethical, cultural, political and institutional factors impede the development of health research and systems in Kurdistan, Iraq, with the ongoing conflict in the surrounding area and inter-dependence of these factors accounting for the failure or unsustainability of any efforts.

### Funding, community needs and policy environment

Emerging from the external environment are a number of conditions that are critical to successful health research capacity strengthening, namely: the availability of funding, meeting community needs and an enabling policy environment. Many frameworks, especially those targeting LMICs, do not take funding into consideration at the early stage of capacity strengthening but rather consider it as a factor that might later influence the sustainability of the intervention [19]. However, in the MENA region, particularly in conflict settings, the availability of funds to strengthen research is highly limited [82], given the above-mentioned shift in priorities to defence and national security along with the lack of research culture. Finding funding opportunities is also problematic knowing that sources of funding for health research is often separate from funding for humanitarian programs and services. Thus, researchers tend to “tailor” their research to be suitable to funding by external agencies regardless of its social impact, thus rendering both funding and donors’ interests the major driving forces of research [83]. Though this is also common in non-conflict settings, its implications are much more significant in conflict where “useful” research that fits with the community needs is urgently needed, especially on the frontline.

Many frameworks consider policy as a key factor for research capacity strengthening. For instance, Bowen and Zwi [38] present a framework of action which is entirely influenced, if not driven, by policy; but they argue that the latter is mainly determined by evidence and thus can be considered in accordance with community needs. Similarly, Cooke [40] highlights the impact of policy on capacity strengthening on all levels of her framework.

Despite the important role that policy plays in driving research in stable settings, it has a secondary role in research capacity strengthening in conflict compared to other factors like funding, partnership with international institutions and the willingness of local populations to participate, all of which contributes to initiatives that have direct impact on research on health. Thus, we argue that while policy is often paralysed in conflict settings, community needs become a contributing factor to research. This is not to exclude the role that governments can play in promoting/hindering research and its capacity strengthening. For instance, health interventions in Lebanon are not strongly monitored by the government where international agencies have established parallel systems in response to the Syrian refugee crisis [84]. Similarly in Iraq, there are limited regulations on the private healthcare sector [85]. On the other hand, research is strongly regulated and monitored in Egypt [86] and Yemen, where any health intervention is highly controlled by armies or militias, depending on the site of the intervention, and is politically instrumentalised [87, 88].

### Assessing existing capacity and needs

The immediate response to most emergency situations is a humanitarian impulse to act, and urgently. However, it is important to identify actual needs and priorities, especially in conflict where human and physical resources are already stretched [89, 90]. Thus, a comprehensive needs assessment to determine the actual gaps in research in conflict settings is crucial and should be considered as part of the planning process for research capacity strengthening [91]. Since such an assessment provides more upfront knowledge regarding research needs, especially on the frontlines, it can also be considered as a tool for evaluating ongoing interventions. As a result, our framework includes a feedback loop from this section to the one above (funding and community needs) so that interventions that meet community needs are prioritised for funding in an attempt to bridge the gap between evidence and practice, a problematic issue even in non-conflict settings [37, 92]. While the quality and quantity of data in conflict is usually limited, recent reports indicate improved data quality in conflict due to, to a large extent, needs assessments and research conducted as part of the humanitarian response [82, 93–95].

### Infrastructure and communication

In spite of its importance, communication is rarely mentioned in the literature as a stand-alone factor for research capacity strengthening as it is mainly considered as one of the core concepts of partnership [96]. However, in conflict settings, communication – even on the local level – is challenging. So, providing access to reliable internet and other communication tools is crucial to conduct rapid and high-quality community-based research and to have effective capacity strengthening on both institutional and individual levels [97–99].

Infrastructure is a basic pillar in any health research capacity strengthening framework. For instance, Baillie, Bjarnholt [36] place resourcing, including infrastructure, at the very bottom of the pillars for research capacity strengthening for public health. In Cooke’s framework, principle number six focuses on “investing in infrastructure” as a specific intervention for successful research capacity strengthening. That said, physical infrastructure is not given much attention compared to other components like project management and annual appraisal [40]. In frameworks focusing on research capacity strengthening in the global North, much emphasis is placed on improving individual skills and governance over physical infrastructure that, despite its importance, is counted as an antecedent [43, 45, 65].

However, infrastructure is a main consideration and often a limiting factor in health research capacity strengthening in conflict settings. The availability of safe and accessible buildings to conduct primary research and training is crucial [49, 84]. Yet, we have seen ample evidence of how healthcare infrastructure has been a target of attacks during conflicts in Syria, Yemen and Iraq in recent years [100]. Hence, considering the challenges of providing safe physical surroundings, distance learning capabilities and communication with other locations is critical. This has been shown to be useful in conflict settings like Yemen where SMS text messaging was used for education and health promotion in a project conducted by UNICEF [101]. Similarly, tele-medicine was successfully used for mentoring medical doctors and providing clinical decision support in intensive care units in different parts of Syria and in operation rooms in Gaza [102, 103].

Similarly, a major entity that should be established and continuously monitored is the Institutional Review Board (IRB) for ethical review of research protocols. IRBs are often not well-prepared to provide rigorous and rapid reviews of health research protocols in conflict settings and with conflict-affected populations. As for the MENA region, there is a large disparity when it comes to the presence of regulatory bodies for ethics and their adherence to international guidelines. For instance, in Lebanon, a unified system of research governance does not exist, and hence research regulation is greatly influenced by the policies of individual institutions and their IRBs [104]. Syria and Iraq also do not have specific guidelines for research ethics but refer to international guidelines like the Helsinki Declaration, and International Ethical Guidelines for Biomedical Research Involving Human Subjects (CIOMS). Palestine and Yemen, however, have no documents that refer to any national or international guidelines for research ethics [105]. Thus there is an urgent need to strengthen the research ethics capacity at both the institutional and individual level in the region by having programs which are similar to Middle East Research Ethics Training Initiative (MERETI) and Salim El-Hoss Bioethics and Professionalism Program (SHBPP) [106].

Language can also be a barrier to conducting research in conflict, especially in the MENA region where along with Arabic *fus’h*a there are 16 other Arabic dialects which differ significantly from each other [107]; not to mention other languages that are widely spoken in MENA like Farsi, Turkish, Hebrew, Kurdish, along with several minority languages [108]. The interaction of local researchers with international partners and the dissemination of research findings also necessitates a knowledge of the English language by local researchers. As a result, infrastructure to mitigate language barriers like translators and presence of adequate language facilities in conflict are indispensable [109]. Despite being a common feature in both general and conflict settings, language barriers can have drastic consequences in conflict as the timely critical local narratives and qualitative data require rapid and accurate accurate interpretation.

In conflict-affected regions, funding, communication, and infrastructure are thus the main limiting factors for capacity strengthening and tend to be cross-cutting and interdependent. Consequently, infrastructure was incorporated into our model in a triangular shape that shows the additive nature of the factors included: physical infrastructure and a safe location is the top requirement for research capacity strengthening which can then feed into more advanced infrastructure building (labs, IT, etc.). The investments needed in infrastructure are usually larger than any single capacity strengthening activity or program, often needing large financial and technological investment, not to mention additional safety and security measures which could be challenging in conflict [49, 110, 111].

### Training, leadership and partnership

Another framework that emerged in the mid-1990s with the *Framework of Levels* is the *Framework of Partnership* that focuses on the need for equitable and effective partnership between donors and beneficiaries or local and international bodies when strengthening capacity, so that sustainable development would eventually be locally owned [76, 112, 113]. Building partnerships is widely adopted in most of the health interventions in LMICs [75, 76, 114, 115], however the level of collaboration between researchers and relief agencies is still underdeveloped and challenging [116, 117]. To overcome this aspect, ELRHA’s Research for Health in Humanitarian Crises (R2HC) programme provides funding for partnerships between academic institutions and humanitarian NGOs. Between 2013 and 2019, seven funding calls have resulted in more than 50 studies being funded. A recent report by ELRHA highlighted the importance of strong collaboration between the different stakeholders involved in a project in a humanitarian setting as it facilitates data collection and thus leads to well-designed and contextualised interventions [118].

For Baillie, Bjarnholt [36], ‘leadership’ and ‘resourcing’ lie at the heart of the capacity strengthening process. In our model, training, leadership, and partnership are major research capacity strengthening activities. Training is part of the individual capacity strengthening process while partnerships takes place at the organisational and institutional levels. Leadership fits in the middle of these two categories. Similar to the research capacity strengthening levels, we modelled training, leadership, and partnerships as a Euler diagram, due to the overlap between these categories.

Farmer and Weston [45] discuss disciplinary diversity in partnerships, something we captured in the model in two ways: partnerships between researchers of different disciplines and those between different academic institutions and practitioner groups (e.g. from NGOs). This is critical to health research capacity strengthening in conflict-affected contexts since knowledge of different disciplines in sciences, social sciences, and medicine are required. Partnerships with practitioners are key in conflict settings as they will often have better access to affected populations [52]. Partnerships therefore help to bridge the gap between research and practice by involving practitioners in primary research.

We also included North/South partnerships in our model, as these can be crucial in many ways, such as providing distance learning, increased lab capacity, training, mentorship, and so on [69]. However, despite the potential benefits of such partnerships, there is a risk of inequality between local and international partners where research topics are specified by the Northern partners and the external funders, along with inequality in the distribution of the benefits of research [96, 119–123]. Endeavouring to overcome these inequities should be at the heart of any partnership.

For leadership, we focused on governance and mentorship. Governance or management is more of an organizational aspect and is necessary to build strong research projects, strengthen partnerships, and manage funding. Mentorship focuses more on the individual side of capacity strengthening and is crucial for increasing the number of young researchers in the pipeline. It can take multiple forms, but primarily serves early career researchers and students. Mentorship is often local, but can take place in North/South partnerships as well. We also note the overlap between mentorship and training: while mentors certainly provide training, there is some training needed for mentors/leaders to be effective [124]. This is an often overlooked aspect of capacity strengthening but can improve relationships between mentors and mentees and increase the number of researchers focusing on health in conflict and establish sustainable working groups [124]. Equally overlooked is reverse mentoring where early career researchers provide new skills and ideas to more established researchers. A great example of the establishment of such a working group is the International Working Group on Reproductive Health, which contributed to capacity strengthening in the MENA region by supporting and creating a research community that included early career researchers and seniors with multidisciplinary backgrounds [50].

Training, meanwhile, is important for expanding the number of researchers and improving their research and language skills. For health research capacity strengthening, it is aimed at two groups: students (undergraduate and postgraduate) and practitioners [125]. This is important in conflict settings where we primarily look to involve practitioners. These individuals may not have prior research training or experience so courses and workshops can be very effective at increasing the prospects and potential of joint projects. The trainings for both students and practitioners should include topics like qualitative and quantitative research skills, research ethics, and data analysis, all of which help in mentoring individuals who wish to conduct research during humanitarian crises [126].

Gender equity in academia is already a challenge even in the most developed countries [127]. As for the MENA region, gender gap is normalized but varies from country to country depending on culture, social norms, policies, and stability [128]. Ongoing conflicts and patriarchy have restricted the progress in women rights and their political roles. Yet following the Arab spring many countries are in a transition state, and deeply-embedded institutional and cultural barriers to gender equity are being questioned and reconsidered. Positive developments regarding gender equity in general, and women in academia in specific, can thus be achieved in the near future [129]. Capacity building interventions should promote gender equity, especially in conflict settings, by training, mentoring and empowering local female researchers to become leaders in the field of health research. Hence, “Gender Equity” as a theme in the framework is expanded to cover all aspects of capacity strengthening at the individual and institutional levels.

However, the extent to which training, partnerships and leadership can be implemented depends highly on the availability of infrastructure and communication which, as mentioned before, can be hindered in conflict unless advanced technology and innovative approaches are used to overcome these challenges.

### Adaptability and sustainability

Sustainability is a crucial component of capacity strengthening, yet is a major challenge. Consequently, it is an essential part of frameworks for health research capacity strengthening, and is considered as part of the feedback loop within these frameworks [5, 37, 40, 41, 43, 130]. Sustainability is usually attained when the newly acquired skills and facilities following a certain intervention are well maintained and put to use, i.e. individual researchers and teams continue to conduct health research with improved quality [131, 132]. However, *“uncertainty is the only certainty there is”* in conflict and thus sustainability is challenged by many factors like funding, scarcity of resources, political and economic instability, the downward spiral of fragility including the collapse of educational and health systems. In conflict, the flight of health workers, researchers and skilled administrators is one of the key barriers to sustainability [133]. For example, thousands of health practitioners have left Syria since 2011, which has led to a severe shortage in health workers, especially in the most severely affected areas of the country, such as Aleppo where 96% of medical doctors had fled by 2014 [134, 135].

In our model, adaptability is purposefully placed before sustainability. In conflict, the whole setting is fragile and subject to changing dynamics. Capacity strengthening should thus align with humanitarian work with respect to preparedness and adaptability to changes, such as through the use of tools that can be set up and dismantled easily, data collection tools that could be used in emergencies like District Health Information Software (DHIS)^1^ and KoBo,^2^ and modifying the content of training to be more suitable to the current situation [136]. One example is MSF’s application that was launched in 2017 and is used by MSF fieldworkers and by organizations like WHO and UNICEF. The application is seen as a tool for training the fieldworkers as it provides the latest medical guidelines [137]. Another example is the MENA Youth Capacity Building in Humanitarian Action (MYCHA) programme, which conducts training to youth from the MENA region. It aims to empower youth in conflict settings by preparing them to be involved in response and conflict resolution and by providing six-month mentorship and support for their own humanitarian projects that they implement within their local contexts. The content of such trainings are regularly refined in order to address the realities faced in the field [138] This element is also the beginning of a feedback loop in our model. Capacity strengthening is a continuous process, and our feedback loop relates back to both the external environment and assessment of needs and priorities.

### Monitoring and Evaluation

Another factor that feeds into the feedback loop is monitoring and evaluation [67]. The targets in our model are aimed at health research in conflict settings including evidence-based practice, auditing, and effective dissemination. Long-term impacts like changing institutional and policy norms to support conducting research with a social impact are also included within the monitoring and evaluation process. Such long-term objectives are in accordance with the strategies addressed in the DFID and Cooke frameworks that consider changing the rules of the game as desired long-term ends for capacity strengthening [40, 41].

In an unstable and high-risk setting, where even daily activities have to be negotiated and adapted, it is difficult to implement monitoring and evaluation activities, and to predict both long-term and short-term impact. Thus, in such settings, real-time evaluation for capacity strengthening, as a tool of continuous improvement and development, is more beneficial compared to the conventional thematic evaluation approach for frameworks of capacity strengthening due to the lack of knowledge and stability [49].

Another question that also arises is whether capacity strengthening in conflict should be considered as an end or as a process to an end [40]. Since long-term objectives are less likely to be achieved in fragile settings, it would be more realistic to consider capacity strengthening, with its standard indicators of dissemination and number of trainings conducted, as an end in the context of conflict when evaluating the intervention.

## Limitations

The search in the literature for frameworks was based on systematic search using key words that align with our definition of capacity strengthening, in a field with inconsistent terminology [139]. Thus, it was challenging to form a search strategy that would uncover all relevant articles and frameworks. This limitation was not unique to this paper, as other recent reviews of capacity building/strengthening literature have noted similar challenges [140, 141]. Similarly, we faced the same challenge when looking for references in Arabic. Therefore, we cannot claim that we identified all of the frameworks available in the literature that focus on capacity strengthening for health research. Another limitation is that the search only included reports and journal articles that are written in English and Arabic and not in other languages of the MENA region, such as French or Turkish.

## Conclusion

The model presented in this work synthesises the different evaluation frameworks for health research capacity strengthening, while paying specific attention to the challenges presented by conflict settings, particularly in the MENA region. It expands on the existing frameworks and connects the broad overview framework used in the literature with more detailed structures. Though any progress achieved in any structural level would influence overall research capacity strengthening, sustained effort at all levels is needed. The model can also be considered as a non-prescriptive reference tool for people involved in research capacity strengthening to evaluate, use, adapt and improve. We have invested our efforts in developing this comprehensive model with an operational focus, developed for a wide audience of stakeholders in mind, from healthcare practitioners and researchers to funding agencies, and across disciplinary divides, encompassing both the medical sciences and the social sciences. It can be further extended to include representative indicators and can be later evaluated by assessing its efficacy for interventions in conflict settings.

## Methods

The framework was synthesized in accordance with the methodology provided by Carroll et al. [142] on “best fit” framework synthesis which provides the ability to design a context-specific model by building on existing models and theories and testing their feasibility, applicability and their fit to evidence from a certain context. Relevant literature written in both Arabic and English, including frameworks and conceptual models, were collected and reviewed through PubMed and the internet search engines Google Scholar and Google. Examples of keywords included are: capacity building; capacity strengthening; health research; framework and conflict. Frameworks, policy papers and reports publicly available on the websites of key funders and international and bilateral organisations were also screened. We only selected documents which described frameworks that meet our definition for capacity strengthening (see Table 1), are widely applicable, and are not intervention specific.

A structured qualitative approach was used to analyse the selected documents to be reduced to their key elements and variables, such as levels, sublevels, external environment, evaluation, and applicability in conflict settings; all of which form the major themes of the proposed framework [143]. Evidence from included studies was also used to feed into existing themes or to form new themes within this framework. The proposed framework, and its themes, are therefore the result of the thematic analysis and contextualized interpretation of evidence from the various frameworks reported in published and grey literature.

## Data Availability

All data generated or analysed during this study are included in this published article.

## Abbreviations

DFID: Department for International Development
DHIS: District Health Information Software
DRC: Danish Refugee Council
GDP: Gross Domestic Product
IDRC: International Development Research Centre
LMIC: Low and Middle-Income Country
LPHA: Lancet Palestinian Health Alliance
LSHTM: London School of Hygiene & Tropical Medicine
MENA: Middle East and North Africa
MSF: Médecins Sans Frontières
MYCH MENA: Youth Capacity Building in Humanitarian Action
NGO: Non-Governmental Organization
NRC: Norwegian Refugee Council
PTSD: Post-Traumatic Stress Disorder
R2HC: Research for Health in Humanitarian Crises
R4HC: Research for Health in Conflict MENA
RECAP: Research Capacity Building and Knowledge
REDCAP-MED: Research Capacity for Public Health in the Mediterranean
SAFOD: Southern African Federation of the Disabled
SRP: SAFOD Research Programme
UN OCHA: United Nations Office for the Coordination of Humanitarian Affairs
UN: United Nations
UNDP: United Nations Development Programme
UNHCR: United Nations High Commissioner for Refugees
UNICEF: United Nations International Children’s Emergency Fund
WHO: World Health Organization

## Footnotes

1 DHIS is an open source software platform for reporting, analysis and dissemination of data for all health programs.

2 The KoBoToolbox is a set of tools for building forms and collecting interview responses built by the Harvard Humanitarian Initiative for use in difficult field settings, such as humanitarian emergencies or post-conflict environments. It is currently hosted and supported by United Nations Office for the Coordination of Humanitarian Affairs (UN OCHA)
